# Structural and Functional Analyses of Type I IFNa Shed Light Into Its Interaction With Multiple Receptors in Fish

**DOI:** 10.3389/fimmu.2022.862764

**Published:** 2022-03-22

**Authors:** Zixuan Wang, Jing Xu, Jianhua Feng, Kaizheng Wu, Kangyong Chen, Zhao Jia, Xiaozhen Zhu, Wenji Huang, Xin Zhao, Qin Liu, Bangjie Wang, Xinhua Chen, Junya Wang, Jun Zou

**Affiliations:** ^1^ Key Laboratory of Exploration and Utilization of Aquatic Genetic Resources, Ministry of Education, Shanghai Ocean University, Shanghai, China; ^2^ International Research Center for Marine Biosciences at Shanghai Ocean University, Ministry of Science and Technology, Shanghai, China; ^3^ National Demonstration Center for Experimental Fisheries Science Education, Shanghai Ocean University, Shanghai, China; ^4^ Key Laboratory of Marine Biotechnology of Fujian Province, Institute of Oceanology, Fujian Agriculture and Forestry University, Fuzhou, China; ^5^ Laboratory for Marine Biology and Biotechnology, Qingdao National Laboratory for Marine Science and Technology, Qingdao, China

**Keywords:** interferon, structure, receptor, antiviral activity, fish

## Abstract

Teleost type I interferons (IFNs) are categorized into group I and II subgroups that bind to distinct receptors to activate antiviral responses. However, the interaction between *ifn* ligands and receptors has not fully been understood. In this study, the crystal structure of grass carp [*Ctenopharyngodon idella* (*Ci*)] IFNa has been solved at 1.58Å and consists of six helices. The *Ci*IFNa displays a typical structure of type I IFNs with a straight helix F and lacks a helix element in the AB loop. Superposition modeling identified several key residues involved in the interaction with receptors. It was found that *Ci*IFNa bound to cytokine receptor family B (CRFB) 1, CRFB2, and CRFB5, and the three receptors could form heterodimeric receptor complexes. Furthermore, mutation of Leu27, Glu103, Lys117, and His165 markedly decreased the phosphorylation of signal transducer and activator of transcription (STAT) 1a induced by *Ci*IFNa in the *Epithelioma papulosum cyprini* (EPC) cells, and Glu103 was shown to be required for the *Ci*IFNa-activated antiviral activity. Interestingly, wild-type and mutant *Ci*IFNa proteins did not alter the phosphorylation levels of STAT1b. Our results demonstrate that fish type I IFNs, although structurally conserved, interact with the receptors in a manner that may differ from mammalian homologs.

## Introduction

Soluble factors with antiviral activities similar to interferon (*ifn*) were reported in fish 50 years ago (1). But it was not until 2003 when the first fish type I *ifn* gene was identified in three fish species including zebrafish (*Danio rerio*) (2), Atlantic salmon (*Salmo salar*) (3), and puffer fish (*Takifugu rubripse*) (4). To date, it is known that teleost fish, like mammals, possess a complex *ifn* system to defend against virus infection. Based on the patterns of cysteines involved in the formation of disulfide bonds, teleost fish type I IFNs can be classified into two subgroups, one containing two cysteine residues (group I) and one containing four cysteine residues (group II) (5, 6), and can be further divided into seven phylogenetic groups, namely, IFNa–f and IFNh (7–12). Group I IFNs consist of IFNa, d, e, and h, while group II IFNs comprise b, c, and f. Recently, type I *ifn* genes have been identified in cartilaginous fish (13), indicating that IFNf may represent the ancestral *ifn* group in jawed vertebrates.

Type I and III IFNs share similar antiviral properties and are believed to have evolved from a common ancestor. Type I *ifn* genes in cartilaginous fish and bony fish contain 5 exons and 4 introns, the same genomic organization to that of type III *ifn* genes in sharks and mammals (9). It is interesting that type III *ifn* genes have not been identified in teleosts. Tetrapod type I *ifn* genes lack introns. However, despite a high sequence diversity, all type I IFNs share a similar structure consisting of 6 alpha helices (A–F) that are arranged in unique up-up-down-down topology (14). Helices A, C, D, and F form an anti-parallel bundle, and loop AB and loop DE are most variable (15). The helices of type I IFNs are long, straight, and arranged parallel to one another (16). The crystal structures of zebrafish IFNφ1 and IFNφ2 have been solved, which are the only available structures for type I IFNs in fish (17). The overall topology of zebrafish IFNφ1 and IFNφ2 is conserved with that of type I and III IFNs in humans. However, it is more similar to that of human type I IFNs than type III due to the structural difference of helix F (17).

Type I IFNs bind to the same heterodimeric receptor to activate cellular responses. The receptor complex consists of IFNAR1 and IFNAR2, both of which belong to the class II cytokine receptor family (18). The domain structure of the extracellular region of IFNAR1 is unique compared to other class cytokine receptors, consisting of 4 fibronectin III subdomains (SD1–4), with SD4 domain adjacent to the membrane and is not involved in ligand binding. It is believed that SD1–2 and SD3–4 may have arisen from domain or gene duplication events that occurred in the ancestor of tetrapods (4, 15). The crystal structure of the ligand and receptor ternary complex reveals in humans that the SD1–3 domains of IFNAR1 form the contact interface with helices B, C, and D of the *ifn* ligands (15, 16). Relative to IFNAR1, IFNAR2 has high binding affinity with IFNs (19). IFNAR2 binds to a contact area involving several key residues in helix A, the AB loop, and helix F. Contrasting with the findings in humans, murine IFNAR1 and IFNAR2 are the high and low binding receptors for type I IFNs (20). This implies that the interaction of *ifn* ligands and receptors may differ among vertebrates.

Fish type I *ifn* receptors differ in several features from their mammalian counterparts. Firstly, fish have multiple orthologs of IFNAR1 and IFNAR2 (21–23). Secondly, fish IFNAR1 orthologs only contain 2 SD domains rather than four SD domains seen in tetrapod counterparts (22, 24). Functional studies have shown that CRFB1 pairs with CRFB5 to form a receptor complex that interacts with group I IFNs, while the CRFB2/CRFB5 form a heterodimeric receptor that is activated by group II type I IFNs (25). Using a gain- and loss-of-function approach, CRFB1 and CRFB5 were shown to be essential for the antiviral activity of IFNφ1 and *ifn* φ4 (group I) in zebrafish. Conversely, zebrafish IFNφ2 and IFNφ3 (group II) elicit an antiviral response through CRFB2 and CRFB5 (25, 26). The usage of distinct receptors by type I IFNs have also been proven in mandarin fish where IFNd and IFNh (group I) preferentially activate the CRFB1/CRFB5 receptor to induce the expression of *ifn*-stimulated genes (ISGs) (12). Despite functional characterization, how fish *ifn* ligands interact with their receptors is still unclear.

STAT1 phosphorylation is central for transduction of type I *ifn*-mediated antiviral response. Upon activation by IFNs, JAK1 and TYK2 are recruited to the cytoplasmic region of IFNAR1 and IFNAR2, facilitating phosphorylation of STAT1 and STAT2. Phosphorylated STAT1 and STAT2 form a complex of IFN-stimulating gene factor 3 (ISGF3), which subsequently translocates into the nucleus to trigger the expression of ISGs (27, 28). The *stat1* gene has been reported in a number of fish species (29–33) and is now known to exist as two copies, *stat1a* and *stat1b*, which are believed to have been duplicated by the teleost-specific whole-genome duplication event. Zebrafish *stat1a* and *stat1b* are located in chro 22 and chro 9, respectively (34, 35). Fish STAT1a contains all five conserved domains required for functioning, while STAT1b lacks the C-terminal transcriptional activation domain (35). Functional studies show that STAT1a transduces signals triggered by IFNs (32, 36, 37). However, the roles of STAT1a and STAT1b in the regulation of *ifn* pathway in different fish species are still under debate (38).

In this work, we solved the crystal structure of grass carp IFNa and determined the interaction with its receptors. Key residues of IFNa involved in contact with receptors were identified and functionally characterized. In addition, the role of STAT1a and STAT1b in mediating *ifn* signaling was investigated.

## Materials and Methods

### Cells and Viruses

*Epithelioma papulosum cyprini* (EPC) cells were maintained at 28°C in a 5% CO_2_ incubator in Dulbecco’s modified Eagle media (DMEM, Gibco) culture medium supplemented with 10% fetal bovine serum (FBS; Gibco) and 1% Pen/Strep (Gibco). HEK293 adherent cells were grown at 37°C in a 5% CO_2_ incubator with DMEM and 1% Pen/Strep. HEK293F cells were maintained at 37°C in an 8% CO_2_ cell shaker (relative humidity greater than 80%) in Expi293™ expression medium (Gibco). Spring viremia of carp virus (SVCV) was kindly provided by Dr. Mingxian Chang, Institute of Hydrobiology, Chinese Academy of Sciences, and propagated in the EPC cells as previously described (39).

### Plasmids

The IFNa sequence of grass carp [*Ctenopharyngodon idella* (*Ci*), ABC87312.1] was obtained from the NCBI database. Plasmid (pUC57-*Ci*IFNa) containing the mature peptide sequence (starting from Cys23) was synthesized by GENEWIZ. The mature peptide of *Ci*IFNa was amplified by PCR using primers listed in **Table 1**. To avoid open reading frame shift, two bases (CT) were added to the 5’ end of the forward primer. The *Ci*IFNa fragment was then cloned into the pET-21d expression vector at the *Nco* I and *Bam* HI sites to obtain pET-21d-*Ci*IFNa. The eukaryotic expression plasmids were synthesized by GENEWIZ, including pcDNA3.4-*Ci*IFNa, pcDNA3.1-SVCVP (SVCV phosphoprotein, NP_116745.1), pcDNA3.1-SVCVN (SVCV nucleocapsid protein, NP_116744.1), pcDNA3.1-SVCVG (SVCV glycoprotein, NP_116747.1), pcDNA3.1-SVCVM (SVCV matrix protein, NP_116746.1), and pcDNA3.1-SVCVL (SVCV polymerase protein, NP_116748.1). The *Ci*IFNa mutant plasmids including pcDNA3.4-L27A, pcDNA3.4-E103A, pcDNA3.4-K117A, and pcDNA3.4-H165A were generated using primers in **Table 1**. The plasmids for detecting phosphorylation of STAT1a and STAT1b (pcDNA3.1-*Dr*H-STAT1a and pcDNA3.1-*Dr*H-STAT1b) were synthesized by GENEWIZ, and details are provided in **Supplementary Figure S1**.

**Table 1:** Information of gene primers.

Primer name	Sequence(5’–3’)	Application
*Ci*IFNa	F: CATGCCATGG **CT**TGCGAATGGCTCGGTCGCTACCGT	Prokaryotic plasmid construction
	R: CGGGATCCTTAACGGCGATTGGCGATGCT	
L27A	F: AGAATGATCAGCAACGAGAGCGCCAGCCTGCTGAAGGAG	Construction of mutant plasmid
	R: GGCGCTCTCGTTGCTGATCATTCTGTATCTGCCCAGCCA	
E103A	F: CAGTGGAACCTGCAGACCGTGGCCCACTTCCTGACCGTGCTGAACAGACAGAGCAGCGAC R: GGCCACGGTCTGCAGGTTCCACTGCACGCTGTTCATGTG	Construction of mutant plasmid
K117A	F: AACAGACAGAGCAGCGACCTGGCCGAGTGCGTGGCTAGA R: AACAGACAGAGCAGCGACCTGGCCGAGTGCGTGGCTAGA	Construction of mutant plasmid
H165A	F: CAGATCAGAAGAGCCGTGAAGGCCCACCTGCAGAGAATG R: GGCCTTCACGGCTCTTCTGATCTGCTCCCAGGCTTGGGC	Construction of mutant plasmid
EPC-IFNa	F: ATGAAAACTCAAATGTGGACGTA R: GATAGTTTCCACCCATTTCCTTAA	qPCR
EPC-Mx	F: GGCTGGAGCAGGTGTTGGTATC R: TCCACCAGGTCCGGCTTTGTTAA	qPCR
EPC-Viperin	F: AGCGAGGCTTACGACTTCTG R: GCACCAACTCTCCCAGAAAA	qPCR
EPC-Isg15	F: CAGCCTTGAGGATGATTCCAG R: TGCCGTTGTAAATCAGTCG A	qPCR
EPC-IRF3	F: GTTTAGAGGGACAATTAACTGGACTA R: GAGGGTCCACTCTTTGAAAATG	qPCR
EPC-IRF7	F: AAAGTCTTCGTCAGCACCAGCG R: CTCTCCGAAGCACAGGTAGATGGT	qPCR
EPC-β-actin	F: CACTGTGCCCATCTACGAG R: CCATCTCCTGCTCGAAGTC	qPCR
SVCV-N-native	F: TCTGCCAAATCACCATACTCA R: CCATCTCCTGCTCGAAGTC	qPCR and PCR
SVCV-G- native	F: ATCATTCAAAGGATTGCATCAG R: CATATGGCTCTAAATGAACAGAA	qPCR and PCR
SVCV-N- plasmid	F: AACACTGCTGATGGAGAGCC R: TCTGCTCACGATTGTTCCCC	PCR
SVCV-P- plasmid	F: ACGAGGAGGGAACAAGCAAG R: GTGCAGTCTGAACTCGCTCT	PCR
SVCV-M- plasmid	F: GAGACACTGGCTACAGCTCC R: TATGTTCCGCTCACGTGCTT	PCR
SVCV-G- plasmid	F: ACACCGGAGAGAACGGAAAC R: CCAGGCTTCTCATCTCGTGG	PCR
SVCV-L- plasmid	F: CGACGAGGAGATCGGAAAGG R: TCGCTCATCACGATAGGCAC	PCR

Forward (F) and reverse (R) primers given in the table were used for sequencing the respective amplicons. Bold text (CT) indicates sequences introduced in the primer to avoid open reading frame shift. Underlined are sequences of restriction enzymes.

PCR, polymerase chain reaction; qPCR, quantitative PCR.

### Virus Infection and Transient Transfection

The EPC cells were inoculated into 12-well cell culture plates and cultured for 12 h in a 28°C/5% CO_2_ incubator. The cells were infected with 200 μl SVCV [multiplicity of infection (MOI)=1 or 0.1], and control cells were treated with MEM and placed in a 22°C/5% CO_2_ incubator for 1 h to allow virus adsorption. One milliliter MEM with 2% FBS was added to the cells. After 24 h, the cells were washed with phosphate buffered saline (PBS) and collected.

The EPC cells and HEK293 cells were seeded in cell culture plates or 25-cm^2^ culture flasks and transfected with plasmids using jetPRIME^®^ transfection reagent (Polyplus) according to the manufacturer’s protocol. Here, 2 µg poly(I:C) (Sigma) was transfected into the EPC cells using the Lipofectamine™ 3000 Transfection Reagent (Invitrogen).

### Quantitative Real-Time PCR

Total RNA was extracted using TRIzol™ Reagent (Invitrogen). cDNA was synthesized using a premix Hifair^®^ II 1st Strand cDNA Synthesis Kit (gDNA digester plus) (Yeasen) according to the manufacturer’s instructions. Quantitative real-time PCR (qPCR) was performed using the Hieff UNICON^®^ qPCR SYBR Green Master Mix (Yeasen) and run on the Light Cycler 384 Real Time PCR System (Roche). Primers used for qPCR analysis are given in **Table 1**. The *β-actin* gene was used as an internal control for normalization of expression.

### Production and Purification of Recombinant *Ci*IFNa Protein in Bacteria

Plasmid pET-21d-*Ci*IFNa was transformed into *Escherichia coli* Rosseta (DE3) competent cells. The cells were induced with 1 mM IPTG in a shaker (170 rpm) at 37°C for 6 h and centrifuged at 4,000 g at 4°C for 30 min. Cell pellet of 5-L cultures was resuspended in 100 ml PBS and homogenized under high pressure (800 bar) (40). The cell lysate was then centrifuged at 8,000 g at 4°C for 10 min to obtain the inclusion bodies. The inclusion bodies were washed twice with washing buffer 1 [50 mM Tris-Cl, 300 mM NaCl, 10 mM EDTA, 0.5% Triton-X100, and 0.1% dithiothreitol (DTT), pH 8.0] and then with washing buffer 2 (50 mM Tris pH 8.0, 100 mM NaCl, 10 mM EDTA, and 0.1% DTT, pH 8.0). The inclusion body pellet was weighed and dissolved in denaturing buffer containing 6 M guanidine chloride, 10% glycerol, 50 mM Tris-Cl, 100 mM NaCl, and 10 mM EDTA at a final concentration of 30 mg/ml. Here, 10 ml protein solution was gradually added into 1-L refolding buffer (100 mM Tris-HCl, 2 mM EDTA, 400 mM L-arginine-HCl, 0.5 mM oxidized glutathione, and 5 mM reduced glutathione, pH 8.0) and stirred at 4°C for 48 h to allow protein to be refolded. The refolded protein was concentrated to 30 ml using a 10-kDa cutoff filter, mixed with 120 ml equilibration buffer containing 20 mM Tris-HCl (pH 8.0) and 300 mM NaCl, and centrifuged at 10,000 g at 4°C for 10 min (41). The protein solution was further concentrated into 5 ml and loaded onto a Superdex 75 column (GE Healthcare) for purification. The protein purity and size were analyzed by sodium dodecyl sulfate-polyacrylamide gel electrophoresis (SDS-PAGE), and the concentration was determined by the bicinchoninic acid (BCA) assay. The protein was stored at -80°C.

### Production and Purification of Recombinant Proteins in HEK293F Cells

The HEK293F cells were cultured in a conical flask containing 25 ml Expi293™ expression medium (Thermo Fisher Scientific) in a cell culture shaker (130 rpm). When the cell numbers reached 3 × 10^6^ cells/ml, cells were transferred to a 50-ml centrifuge tube and centrifuged at 800 rpm for 10 min. The cells were resuspended with 25 ml fresh culture medium and placed in a 125-ml flask at a concentration of 3 × 10^6^ cells/ml. The cells were transfected with plasmid using ExpiFectamine™ 293 Reagent (Thermo Fisher Scientific) according to the manufacturer’s instructions. Briefly, 1.5 ml Opti-MEM™ I reduced serum medium (Thermo Fisher Scientific) was mixed with 25 µg of pcDNA3.4-L27A, pcDNA3.4-E103A, pcDNA3.4-K117A, or pcDNA3.4-H165A plasmid. Then, a solution containing 80 µl ExpiFectamine™ 293 Reagent (Thermo Fisher Scientific) and 1.4 ml Opti-MEM™ I reduced serum medium was prepared and mixed by pipetting. After incubation at room temperature for 5 min, the above two solutions were mixed, left at room temperature for an additional 20 min and added to the flasks containing the 293F cells. The cells were cultured in a CO_2_ shaker (130 rpm) for 18 h, and 150 μl enhancer 1 and 1.5 ml enhancer 2 were added. At day 4, culture media were collected for purification of recombinant proteins using HisTrap™ HP affinity columns (Cytiva). Purified proteins were verified by SDS-PAGE and Western blotting. The protein concentration was determined as described above.

### Crystallization and Data Processing

The *Ci*IFNa protein purified from bacteria was concentrated to 4 mg/ml and 8 mg/ml in a buffer containing 10 mM Tris (pH 8.0) and 300 mM NaCl for crystallization. Sparse matrix screen kits such as Index, Classic (1–4), Crystal Screen I/II, Peg/Ion Screen, Peg/Ion 2 Screen, and Crystal Screen Cryo I/II (Hampton Research) were used to screen for the crystallization conditions. The protein solutions were mixed with reservoir buffer at a 1:1 ratio and added to the crystallization plates using the sitting-drop vapor diffusion technique at 277.15 K. Crystals appeared after 4 days using the Classic 3 (No.1) kit. Diffraction data on the *Ci*IFNa crystals were collected on beamline BL17U at wavelengths of 0.97923 Å with an ADSC 315 CCD detector at the Shanghai Synchrotron Radiation Facility (SSRF) (38). The *Ci*IFNa crystal diffracted to 1.58 Å resolution. In each case, the crystals were first soaked in reservoir solution containing 15% glycerol as cryoprotectant for a few seconds and then flash-cooled in a stream of gaseous nitrogen at 100 K. The collected intensities were indexed, integrated, corrected for absorption, scaled, and merged using the HKL3000 software package. Please refer to Wang et al. (42) for data refinement, structure determination, and analysis. The crystal structure has been deposited in the Protein Data Bank (PDB) (http://www.pdb.org) under accession number 7WKH. Structure-based sequence alignment adopts was performed using the ESPript program (https://espript.ibcp.fr/ESPript/ESPript/) (43).

### Co-Immunoprecipitation

HEK293 cells were seeded in 25-cm^2^ culture flasks overnight and transfected with 5 μg of plasmid DNA. At 24 h, medium was removed and the cell monolayer was washed with PBS (pH 7.4, Gibco) and lysed in radioimmunoprecipitation assay (RIPA) lysis buffer [50 mM Tris (pH 7.4), 150 mM NaCl, 1% NP-40, 0.5% sodium deoxycholate, 0.1% SDS, sodium orthovanadate, sodium fluoride, EDTA, leupeptin, 150 mM NaCl, 1 mM EDTA, 1 mM NaF, 1 mM sodium orthovanadate] (Beyotime) containing protease inhibitor cocktail (CWBio) on a rocker platform at 4°C for 30 min. The cell lysate was centrifuged at 12,000 rpm at 4°C for 15 min, and the supernatant was collected. Eighty microliters of lysate supernatant was mixed with 20 µl of 5× SDS sample buffer and incubated with 30 µl of Protein A/G Agarose Resin (Yeasen) to remove background. Then, the corresponding primary antibody was added and incubated at 4°C overnight with constant agitation (dilution ratio according to antibody specification). One hundred microliters of 50% Protein A/G Agarose Resin (Yeasen) was added. After incubation at room temperature for 1 h, immunoprecipitated proteins were collected by centrifugation at 2,500 g for 3 min at 4°C, washed three times with ice-cold PBS, resuspended in 80 µl of 2× SDS-PAGE sample loading buffer, and subjected to 12% SDS-PAGE and Western blotting.

### Immunoblotting

Protein samples were separated by 12% SDS-PAGE and transferred to polyvinylidene difluoride membranes using a semi-dry transfer method (BioRad). The membrane was blocked with TBS buffer containing 5% skimmed milk for 1 h and incubated with the primary antibody [diluted 1:1,000 (v/v)] at 4°C overnight. After washing with TBS-T (TBS containing 0.1% Tween 20) buffer for 3 × 5 min, the membrane was then incubated with the IRDye^®^ 800CW Goat anti-Mouse IgG Secondary Antibody [1:10,000 dilution (v/v), Odyssey] or IRDye^®^ 800CW Goat anti-Rabbit IgG Secondary Antibody at 4°C for 1 h, washed with TBS-T buffer for 3 × 5 min, and photographed under the Odyssey CLx image system (Odyssey).

### Plaque Formation Assay and Crystal Violet Staining

The wild-type and mutant proteins (10 ng/ml) were added to the EPC cells in a 6-well plate. After 6 h, the cells were infected with SVCV (MOI = 1), and the medium was replaced with 1 ml MEM containing 2% FBS. After 24 h, cell culture medium was collected and diluted a series of 10-fold for infection experiments. Briefly, the EPC cells were seeded in 12-well plates and incubated with the diluted culture medium for 1 h. Medium was then removed from the wells, and cells were overlaid with 1 ml MEM containing 2% FBS for 48 h. Cells were fixed for crystal violet staining, and plaques were counted manually.

### Statistical Analysis

The statistical analysis was performed using one-way analysis of variance (ANOVA) with Dunnett’s *post-hoc* test (SPSS package 2.0, SPSS Inc., Chicago, USA). P < 0.05, P < 0.01, or P < 0.001 is considered significant.

## Results

### *Ciifna* Is Induced by SVCV and Poly(I:C) but Inhibited by SVCV Proteins

**Figure 1:**
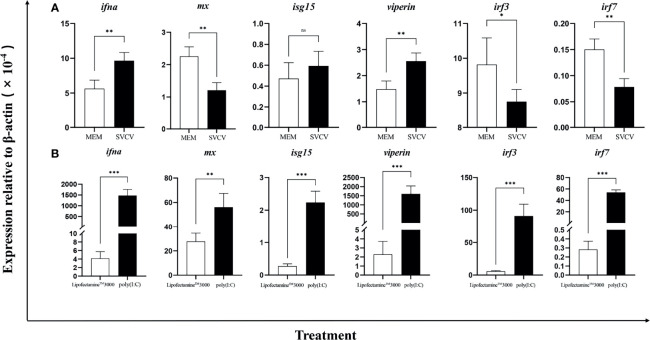
*Ifna* was induced by SVCV **(A)** and poly(I:C) **(B)**. The EPC cells were infected with SVCV (MOI = 1) and transfected with poly(I: C). After 24 h, expression of *ifna*, *mx*, *isg15*, *viperin*, *irf3*, and *irf7* was analyzed by qPCR. Data are shown as mean + SEM (N = 4). *P < 0.05, **P < 0.01, or ***P < 0.001 is considered significant. ns, no significant difference.

To examine the *Ciifna* response to viral infection, the EPC cells were infected with SVCV (MOI = 1) for 24 h. The expression of *svcv-g* and *svcv-n* genes were analyzed by qPCR, confirming that the *svcv-g* and *svcv-n* genes were detected in the infected cells (**Supplementary Figure S2**). We analyzed the expression of *Ciifna* and a panel of several antiviral genes and found that *Ciifna*, interferon stimulated gene (*isg*) 15 and *viperin* were induced, while myxovirus resistance (*mx*), interferon regulatory factor (*irf*) 3, and *irf7* were downregulated. Fish cells are known to express Toll-like receptor 3 (TLR3) that senses poly(I:C), a synthetic double-stranded (ds) RNA analog of virus, to activate an *ifn* response (39). Therefore, we transfected poly(I:C) into the EPC cells. We found that the expression levels of *Ciifna* and *viperin* were markedly increased by 354- and 700-fold, respectively **(** **Figure 1** **)**.

**Figure 2:**
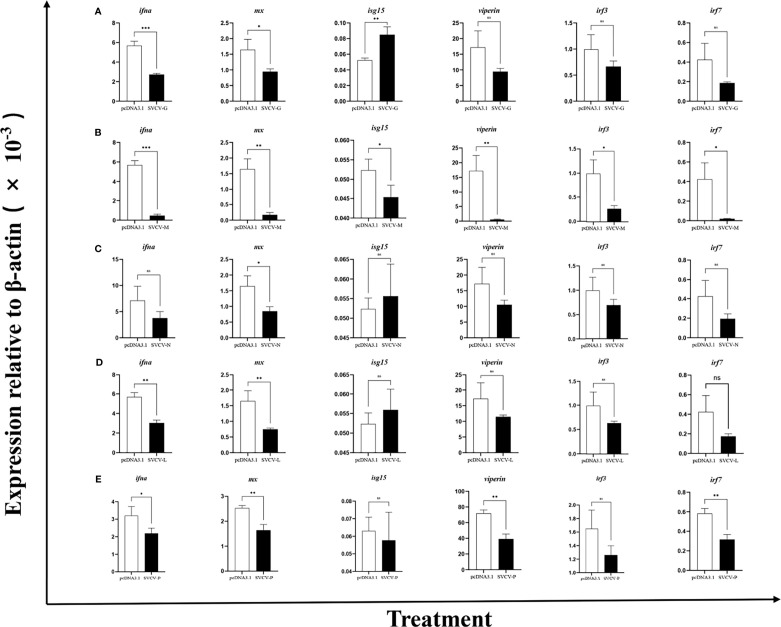
Overexpression of viral proteins downregulates *Ci*IFNa expression. The EPC cells were transfected with plasmids expressing SVCV-G **(A)**, SVCV-M **(B)**, SVCV-N **(C)**, SVCV-L **(D)** or SVCV-P **(E)**. After 24 h, the expression of *ifna*, *mx*, *isg15*, *viperin*, *irf3*, and *irf7* was analyzed by qPCR. Data are shown as mean + SEM (N = 4). *P < 0.05, **P < 0.01, or ***P < 0.001 is considered significant. ns, no significant difference.

Next, we investigated the effects of five individual SVCV proteins on the expression of *Ciifna* and *isgs* **(** **Supplementary Figure S3** **)**. For this, the EPC cells were transfected with plasmid expressing SVCV phosphoprotein (SVCV-P), nucleocapsid protein (SVCV-N), glycoprotein (SVCV-G), matrix protein (SVCV-M), or polymerase protein (SVCV-L). After 24 h, we observed that *Ciifna*, *mx*, *viperin*, *irf3*, and *irf7* were downregulated by all 5 viral proteins except that the *irf7* expression was not affected by SVCV-L **(** **Figure 2** **)**. It must be noted that the *isg15* expression was modulated differentially by viral proteins, with upregulation by SVCV-G, SVCV-N, and SVCV-L but downregulation by SVCV-M and SVCV-P.

#### The Topological Structure of *Ci*IFNa Is Conserved

**Figure 3:**
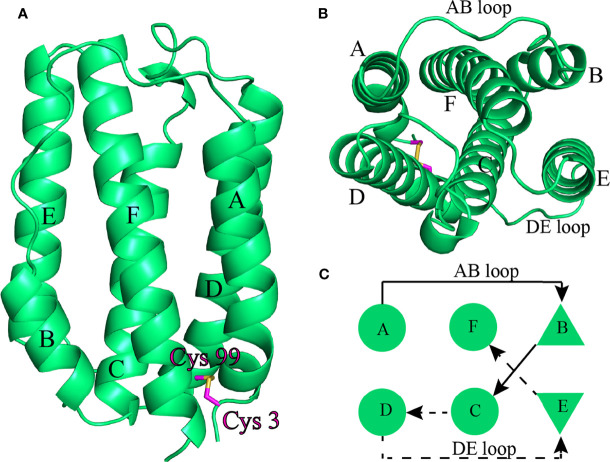
The overall structure of *Ci*IFNa. The structure is shown in cartoon representation and colored in lime green. **(A)** Ribbon structure of dimer. **(B, C)** Ribbon structure **(B)** and schematic diagram **(C)** of monomer. The helix A–F, AB Loop, and DE loop are indicated. The disulfide bond is shown in stick. Solid and broken arrows indicate front and behind positions, respectively.

The crystal structure of *Ci*IFNa was determined at 1.58 Å resolution with space group C121 by molecular replacement. The final refinement of the structure generated Rwork/Rfree factors of 0.1799/0.2251. Details of data collection, phasing, and refinement are given in **Table 2**. In this structure, only one molecular was found. As expected, the structure reveals a typical type I *ifn* architecture of 6 structural elements, denoted A through F (**Figure 3**). The helices A, C, D, and F form an anti-parallel four-helix bundle, which are arranged in an up-up-down-down orientation. The helix F is straight, which is the hallmark of type I *ifn* (15). One disulfide bond formed by Cys3-Cys99 is present in the *Ci*IFNa, linking the N terminal region with helix D, and is analogous to the disulfides found in other vertebrates’ type I *ifn* (15).

**Table 2:** Data collection and refinement statistics (molecular replacement).

	Crystal Data
**Data collection**	*Ci*IFNa
Space group	C121
Cell dimensions	
*a*, *b*, *c* (Å)	84.617, 32.767, 56.762
α, β, γ (°)	90, 102.21, 90
Resolution (Å)	27.75–1.58
Wavelength	0.97923 Å
Beamline	BL18U1
	
**Refinement**	
Resolution (Å)	35.3–1.35
No. reflections	1113
R_work_/R_free_	0.1799/0.2251
No. atoms	919
Protein	100
Ligand/ion	
Water	125
B-factors	17.0
R.m.s. deviations	
Bond lengths (Å)	0.012
Bond angles (°)	1.718
**Ramachandran statistics**	
Preferred regions (%)	100
Allowed regions (%) Outlier (0.00%)	0.00 0.00

Values in parentheses are for highest-resolution shell.

#### The AB Loop of *Ci*IFNa Lacks the Characteristic Helix Element

**Figure 4:**
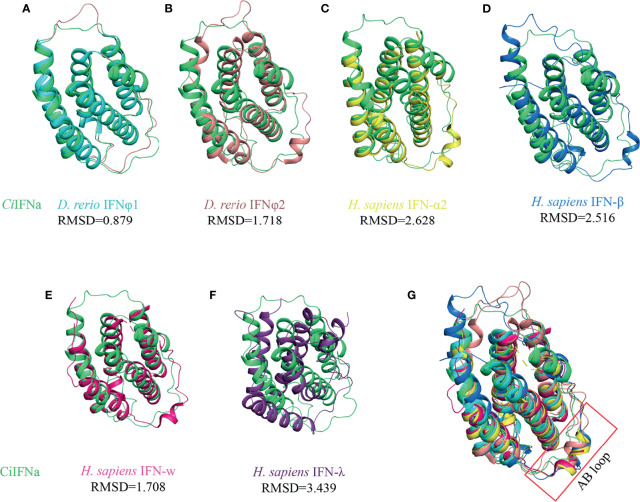
Structural comparison of *Ci*IFNa with type I and III IFNs. All the structures are shown in cartoon. The CiIFNa was superposed with zebrafish IFNφ1 **(A)**, zebrafish IFNφ2 **(B)**, human IFN-α2 **(C)**, human IFN-β **(D)**, human IFN-w **(E)** and human IFN- λ **(F)**. The differences of AB loop are indicated **(G)**. The structure data of zebrafish IFNφ1 (PDB: 3piv) and IFNφ2 (PDB: 3piw), and human IFN-α2 (PDB: 3se3), IFN-β (PDB: 1au1), IFN-w (PDB: 3se4) and IFN-λ (PDB: 3hhc) were retrieved from the PDB database. The structures of IFNs are shown in different colors.

The structure of *Ci*IFNa was compared with that of known type I and III members including human *ifn*-α2, *ifn*-β, *ifn*-w, and *ifn*-λ and zebrafish IFNφ1 and IFNφ2 by superimposition analysis. It is apparent that *Ci*IFNa has the most structure similarity with zebrafish IFNφ1 (fish group I type I) with an root mean square deviation (RMSD) value of 0.879 (**Figure 4A**). Consistently, it has relatively lower RMSD values with other members of type I IFNs such as zebrafish *ifn* φ2 (1.718), human *ifn*-α2 (2.628), *ifn*-β (2.628), and *ifn*-w (1.708) than human *ifn*-λ (3.439) (**Figures 4B–F**). Structure alignment shows that the AB and DE loop of type I IFNs are highly variable. In human *ifn*-w and *ifn*-α2, the AB loop is composed of 17 amino acids and contains an extra helix element (**Figure 4G**). In contrast, the AB loop of *Ci*IFNa and zebrafish IFNφ1 is shorter, containing 14 and 10 aa, respectively. Notably, no helix element is present in the AB loop of *Ci*IFNa (**Figure 4G**).

#### The Key Residues for the Interaction of Type I IFNs With Receptors Are Not Conserved in *Ci*IFNa

**Figure 5:**
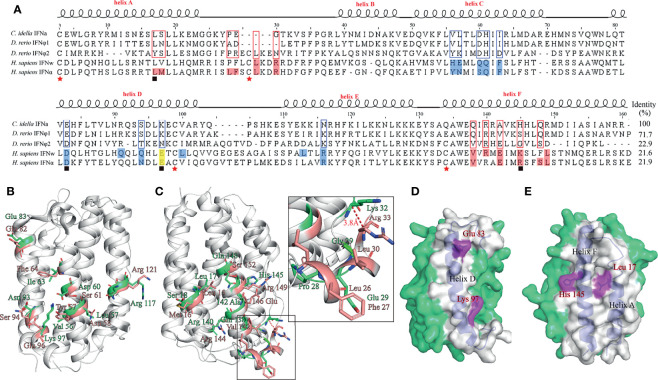
Prediction of key residues of *Ci*IFNa in the interaction with receptors. The corresponding residues for the interaction of IFNs with IFNAR1 and IFNAR2 in *Ci*IFNa. **(A)** Sequence alignment of *Ci*IFNa with zebrafish and human IFNs. Residues for the interaction of human IFNw and IFNα2 with IFNAR1 are colored marine, and those with IFNAR2 are colored salmon, and the corresponding residues in fish IFNs are boxed. Cysteines are indicated by stars. The residues used for mutation are indicated by solid boxes below the alignment. **(B, C)** Prediction of residues for receptor interaction in the *Ci*IFNa. Sticks colored in lime green and salmon represent the residues in the *Ci*IFNa and human IFN-α2, respectively. **(D, E)** The positions of selected residues for mutation.

Human type I *ifn* ternary complex (*ifn*-α2/IFNAR1/IFNAR2 and *ifn*-w/IFNAR1/IFNAR2) shows that the aa residues of IFNs interacting with IFNAR1 are distributed in helices C, D, and E, and those residues in contact with IFNAR2 are placed in helices A/F and AB loop. *Ci*IFNa shares a low sequence identity with human *ifn*-α2 (21.9%) and IFN-w (21.6%), and the residues aligned with those involved in the receptor interaction are poorly conserved (**Figure 5A**). Asp82^hIFNα2^, Glu96^hIFNα2^, and Arg120^hIFNα2^ interact with IFNAR1 *via* the hydrogen bonds, especially Arg120^hIFNα2^ forms a hydrogen bond network, and are substituted by residues Glu83, Lys97, and Asn126 in *Ci*IFNa, respectively. In addition, Phe64^hIFNα2^ involved in the hydrophobic interaction with IFNAR1 is aligned with hydrophobic Ile67*^Ci^* ^IFNa^ (**Figure 5B**).

IFNs bind to IFNAR2 principally through the AB loop, in which arginine (e.g., Arg35^hIFNw^ or Arg33^hIFNα2^) is considered to be the most single residue contributing to the interaction between *ifn* ligands with IFNAR2. In *Ci*IFNa, this position is occupied by glycine. Leu15^hIFNα2^ and Met16^hIFNα2^ form one hydrophobic cluster in human type I *ifn*, and interestingly, the residue corresponding to Leu15^hIFNα2^ is conserved in *Ci*IFNa (Leu17). His145*^Ci^* ^IFNa^, which is also a basic residue and equivalent to Arg149^hIFNα2^, is located in helix F and was predicted to be required for the interaction with IFNAR2 (**Figure 5C**). Based on the comparative analysis, we reasoned to select two key residues potentially engaging in the interaction with IFNAR1 (Glu83*^Ci^* ^IFNa^ and Lys97*^Ci^* ^IFNa^) and IFNAR2 (Leu17*^Ci^* ^IFNa^ and His145*^Ci^* ^IFNa^) for further functional characterization (**Figures 5D, E** **)**.

### *Ci*IFNa Interacts With CRFB1, CRFB2, and CRFB5

**Figure 6:**
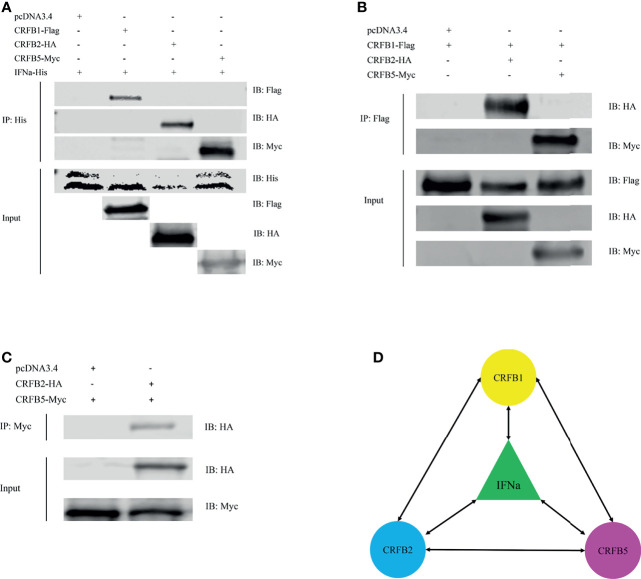
*Ci*IFNa interacts with CRFB1, CRFB2, and CRFB5. The HEK293 cells were transfected with the respective plasmids (2.5 μg each). After 24 h, cell lysates were immunoprecipitated (IP) with antibody agarose resin. The immunoprecipitates and cell lysates were analyzed by immunoblotting (IB) with the anti-His, anti-Flag, anti-HA, or anti-Myc Abs. **(A)** Interaction of CiIFNa with CRFB1, CRFB2 and CRFB5. **(B)** Interaction of CRFB1 with CRFB2 and CRFB5. **(C)** Interaction between CRFB2 and CRFB5. **(D)** Diagram description of interactions between CiIFNa and receptors. Bidirectional arrows indicate interaction. "+"=transfected, "-"=not transfected.

The interaction between fish IFNs and the receptors has not been fully elucidated. Known fish type I *ifn* receptors include CRFB1, CRFB2, and CRFB5 (20, 21). CRFB5 is an ortholog of IFNAR1 in mammals, while CRFB1 and CRFB2 are equivalent to mammalian IFNAR2. Previous studies have shown that fish group I (2 cysteine containing) and group II (4 cysteine containing) type I IFNs interact with different receptor complexes for signaling. While CRFB5 is the common receptor chain shared by all type I IFNs, CRFB1 and CRFB2 have been shown to interact with group I and II IFNs, respectively. In this study, we sought to determine the interaction of *Ci*IFNa with CRFB1, CRFB2, and CRFB5 by co-immunoprecipitation (co-IP). The HEK293 cells were cotransfected with IFNa-His and CRFB1-Flag, CRFB2-HA, or CRFB5-Myc. It was shown that IFNa-His could be immunoprecipitated with CRFB1-flag, CRFB2-HA, and CRFB5-Myc, indicating that IFNa proteins could form a complex with CRFB1, CRFB2, and CRFB5 **(** **Figure 6A** **)**. The observation that *Ci*IFNa bound to CRFB2 is unexpected since previous studies show that *Ci*IFNa interacts with CRFB1 and CRFB5 but not CRFB2. Furthermore, we established the partnerships of the three receptors. We found that the CRFB1-flag immunoprecipitated with CRFB2-HA and CRFB5-Myc **(** **Figure 6B** **)**, and so did CRFB2-HA with CRFB5-Myc **(** **Figure 6C** **)**, confirming the association among the three receptors. Taken together, it can be concluded that *Ci*IFNa binds to CRFB1, CRFB2, and CRFB5, and the three receptors are capable of forming heterodimer or multimer receptor complex (**Figure 6D**).

### Wild-Type *Ci*IFNa and Mutants Have a Similar Binding Affinity With Receptors

**Figure 7:**
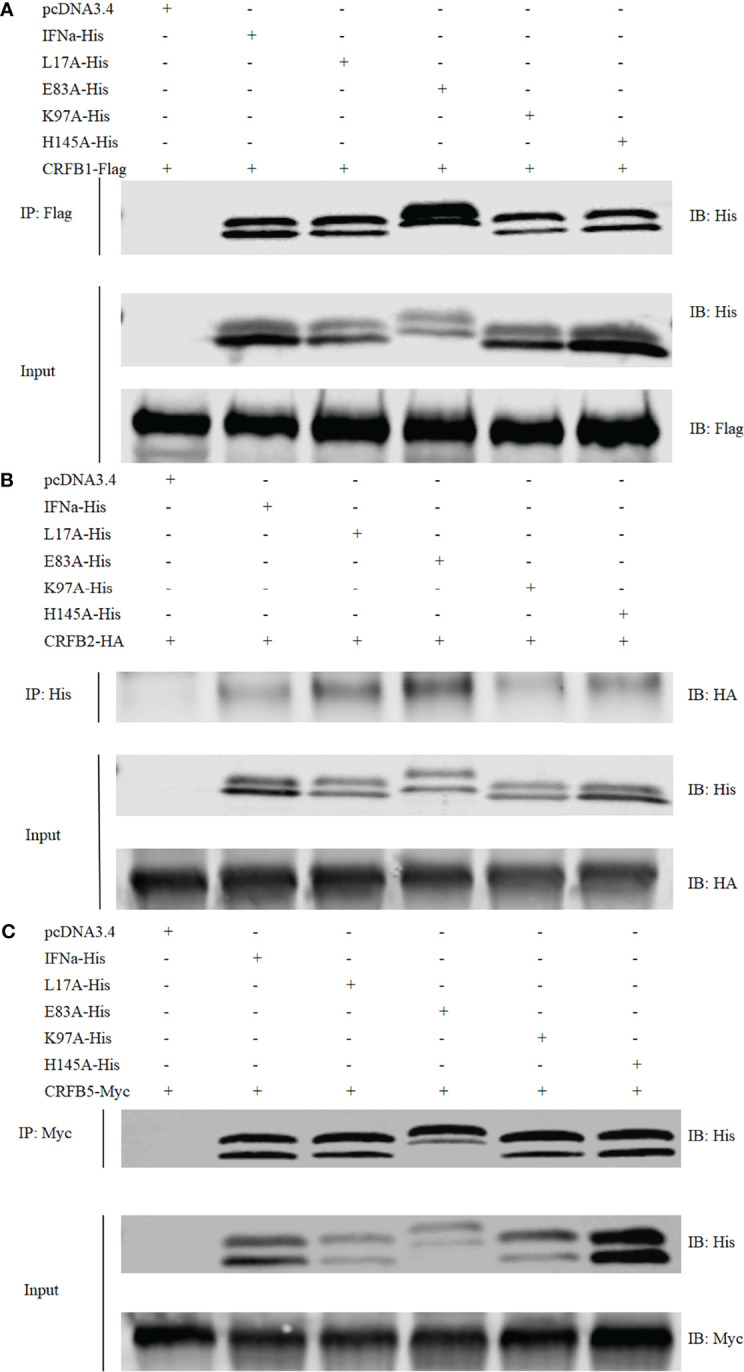
*Ci*IFNa and mutants binds to CRFB1, CRFB2, and CRFB5. The HEK293 cells were transfected with respective plasmids (2.5 μg each). After 24 h, cell lysates were immunoprecipitated (IP) with antibody agarose resin. The immunoprecipitates and cell lysates were analyzed by immunoblotting (IB) with the anti-His, anti-Flag, anti-HA, or anti-Myc Abs. **(A–C)** Interaction of CiIFNa and mutants with CRFB1 **(A)**, CRFB2 **(B)** and CRFB5 **(C)**. "+"=transfected, "-"=not transfected.

Crystal structural analysis identified several residues in the *Ci*IFNa that are potentially involved in contact with the receptors. To evaluate their impact on the binding with receptors, we mutated key residues of *Ci*IFNa based on the structural analysis and constructed expression plasmids for co-IP assay. Structure analysis showed that E103 and K117 are located in the contact interface with IFNAR1/CRFB5, while L27 and H165 are key residues for binding to IFNAR2/CRFB1/2 (**Figure 5**). We selected these residues for mutation to determine that they are on the receptor binding, signaling, and antiviral activity. The wild-type *Ci*IFNa and mutant plasmids were cotransfected with CRFB1-Flag, CRFB2-HA, or CRFB5-Myc into the EPC cells. Western blotting detected two protein bands of approximately 20 and 23 kDa for the wild-type *Ci*IFNa and all mutants (**Figure 7**). Of note, the size of E103A increased slightly compared with the wild-type *Ci*IFNa. Interestingly, co-IP assay revealed that E103A had a higher binding affinity with CRFB1 and CRFB2 than that of the wild-type *Ci*IFNa **(** **Figure 7A** **)**. However, E103A and wild-type *Ci*IFNa displayed a similar binding affinity with CRFB5. Other mutants appeared to have a similar binding affinity with three receptors relative to wild-type *Ci*IFNa.

### *Ci*IFNa Mutants Decrease Phosphorylation of STAT1a

**Figure 8:**
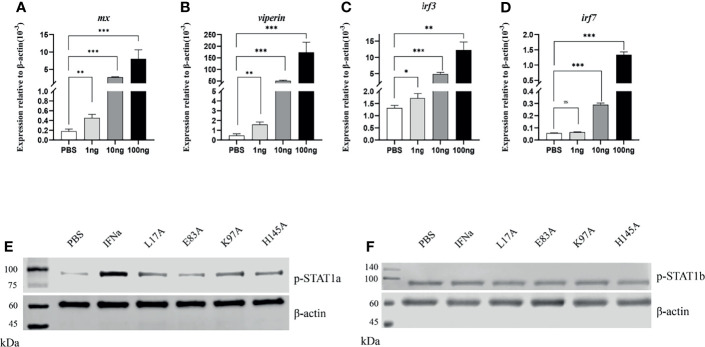
IFNa mutants decreases the phosphorylation of STAT1a but not STAT1b. The EPC cells were stimulated with IFNa derived from HEK293F cells for 6 h. The expression of *mx* **(A)**, *viperin* **(B)**, *irf3* **(C)** and *irf7* **(D)** was analyzed by qPCR. Data are shown as mean + SEM (N = 4). *P < 0.05, **P < 0.01, or ***P < 0.001 is considered significant. The EPC cells were transfected with pcDNA3.1-*Dr*H-STAT1a or pcDNA3.1-*Dr*H-STAT1b plasmid and stimulated with recombinant wild-type and mutant IFNa proteins derived from HEK293F cells, respectively. Phosphorylation of STAT1a **(E)** and STAT1b **(F)** was analyzed by Western blotting. ns, no significant difference.

To evaluate the biological activity of *Ci*IFNa and mutants, the recombinant proteins with a 6 His-tag at the C terminus were produced in the HEK293 cells and purified using affinity chromatography. The purified proteins were validated by Western blotting using 6 His antibody (**Supplementary Figure S5**). To validate the antiviral activity, we stimulated the EPC cells for 6 h with 1, 10, and 100 ng/ml of wild-type *Ci*IFNa protein. We found that the expression of *mx*, *viperin*, *irf3*, and *irf7* was significantly increased in the cells treated with all the three doses (except that *irf7* was unaffected by 1 ng/ml) **(** **Figure 8** **)**. Moreover, a dose-dependent manner was apparent. The results indicate that the *ifn* proteins produced were biologically active.

The type I *ifn*-mediated cellular signaling is coordinated by STAT1 and STAT2. Upon activation of *ifn* receptors by ligands, STAT1 and STAT2 are phosphorylated and form ISGF3 complex with IRF9, which is shuttled into the cell nucleus to trigger the expression of ISGs involved in immune response against viral infection. In teleost, two duplicated copies of *stat1*, namely, *stat1a and stat1b*, are present. Currently, antibodies are unavailable for detecting phospho-STAT proteins in fish, especially for distinguishing the phosphorylation of STAT1a and STAT1b. Therefore, to evaluate the phosphorylation of fish STAT1a and STAT1b, we constructed hybrid zebrafish and human hybrid plasmids (referred to as pcDNA3.1-*Dr*H-STAT1a or pcDNA3.1-*Dr*H-STAT1b) in which zebrafish Tyr 679-Glu717 (STAT1a) and Tyr 683-Ala724 (STAT1b) covering Tyr701 were replaced by the corresponding human STAT1 sequence **(** **Supplementary Figure S1** **)**. In this way, antibodies against human STAT can be used for Western blotting. The EPC cells were transfected with pcDNA3.1-*Dr*H-STAT1a or pcDNA3.1-*Dr*H-STAT1b for 24 h and stimulated with PBS (control) and 10 ng/ml wild-type and mutant *Ci*IFNa proteins purified from HEK293F cells for 10 min **(** **Supplementary Figure S5** **)**. Compared with PBS, all the proteins were shown to increase STAT1a phosphorylation. However, the mutants induced lower levels of STAT1a phosphorylation than the wild-type *Ci*IFNa, with the lowest induction detected for E103A **(** **Figure 8E** **)**. Interestingly, wild-type *Ci*IFNa and mutants did not alter the phosphorylation levels of STAT1b **(** **Figure 8F** **)**.

### Mutants E103A and H165A Exhibit Reduced Antiviral Activity

**Figure 9:**
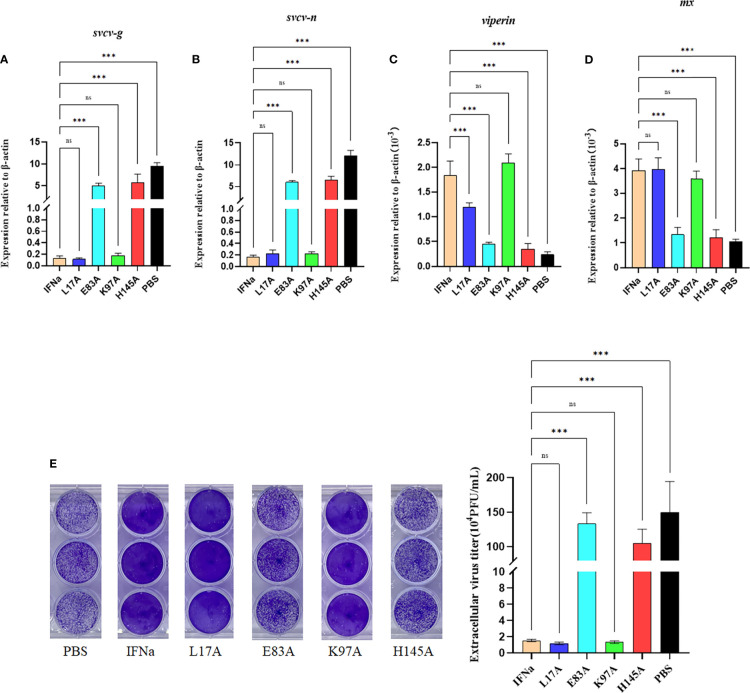
Mutation of E103A and H165A reduces the antiviral activity. The EPC cells were incubated with wild-type IFNa, L27A, E103A, K117A, or H165A proteins for 6 h and then infected with SVCV (MOI = 0.1). After 24 h, total RNA was extracted for analysis of the mRNA levels of *svcv-g* **(A)**, *svcv-n* **(B)**, *viperin* **(C)**, and *mx* **(D)**. Data are shown as mean + SEM (N = 4). ***P < 0.001 is considered significant. ns, no significant difference. The EPC cells were incubated with wild-type IFNa, L27A, E103A, K117A, and H165A proteins for 6 h and then infected with SVCV (MOI = 1). Culture media from infected EPC cells were collected for plaque formation assay using EPC cells. Experiments were performed three times with similar results **(E)**.

We further assessed the antiviral activity of the *Ci*IFNa mutants in EPC cells. The EPC cells were treated with 10 ng/ml of wild-type *Ci*IFNa or mutant proteins for 6 h and then infected with SVCV (MOI = 0.1) for 24 h. The expression of *svcv-g* and *svcv-n* was analyzed. Compared with the wild-type *Ci*IFNa-treated cells, the expression levels of *svcv-g* and *svcv-n* were markedly higher in E103A- and H165A-treated groups **(** **Figures 9A, B** **)**. Consistently, the antiviral genes such as *viperin* and *mx* were markedly inhibited **(** **Figures 9C, D** **)**, and virus titers were higher relative to the cells stimulated with wild-type *Ci*IFNa **(** **Figure 9E** **)**. These results indicate that E103 and H165 are critical for the antiviral activity of *Ci*IFNa. In contrast, the L27A and K117A mutants showed similar antiviral effects with the wild-type *Ci*IFNa.

## Discussion

In this study, we have solved the crystal structure of grass carp IFNa. As expected, it displays typical structure conformation of type I IFNs in other vertebrates, consisting of six helices. Consistently with the crystal structure of type I IFNs, helix F of *Ci*IFNa helix is straight, which is a characteristic feature of type I IFNs and contrasts with the bent F helix of other members of class II helical cytokine family (17). The structure of *Ci*IFNa is highly similar to that of zebrafish IFNφ1 with an RMSD value of 0.879 Å. This is expected that they share high sequence identity (79%), and both belong to the group I type I IFNs (22, 25). However, the AB loop displays significant diversity in the primary sequence, in line with the structure of human *ifn*-α2 and *ifn*-w (15). The AB loop has been shown to be the main interface to contact IFNAR2, which is a high-affinity receptor chain of human type IFNs (15). This suggests that the AB loop is vital for ligand/receptor binding and plays a role in the competition between the receptor complex and other type *ifn*s. One noticeable feature of *Ci*IFNa and zebrafish IFNφ1 is that they lack the helix element in the AB loop compared with human *ifn*-α2 and *ifn*-w. The Arg35^hIFNw^ or Arg33^hIFNα2^ in the AB loop contributes greatly to the interaction with IFNAR2 and is substituted by glycine in the *Ci*IFNa. However, in the corresponding position of 3.8 Å near Arg33^hIFNα2^, basic Lys32 is present in the *Ci*IFNa. We speculate that Lys32 could play an important role in the interaction with the receptor. There are some noticeable differences between fish and mammalian IFNARs: first, fish CFRB5 has 2 SDs in the extracellular region rather than 4 in tetrapod IFNAR1. It is not clear what is the functional significance to have 4 SDs for the tetrapod IFNAR1. It is tempting to hypothesize that 4 SDs may increase the area of contact interface to facilitate receptor sharing by multiple *ifn* ligands. Secondly, there are two duplicated copies of IFNAR2 (24). These structural differences imply that fish type I *ifn*s interact with their receptors in a manner different from their mammalian counterparts. Solving the structure of fish *ifn*/receptor complex will provide answers to this issue.

Type I IFNs belong to the class II helix cytokine family and possess multiple immune functions in antiviral defense. They are induced by viral pathogen-associated molecular patterns (PAMPs) and viruses and can also be counteracted by viral components. In this study, we found that poly(I:C), a synthetic dsRNA, when transfected into EPC cells, induced *Ci*IFNa expression. This is expected because the Toll like receptor 3 (TLR3) sensing dsRNA is known to exist in fish (45). In addition to TLR3, other pattern recognition receptors (PRRs) such as TLR7/8, RIG-1, and MDA5 are also present in fish. We observed the opposite effects of SVCV and poly(I:C) on the expression patterns of *mx*, *irf3*, and *irf7*. This suggests that SVCV could suppress the expression of genes involved in *ifn* production and antiviral defense. Interestingly, overexpression of all five SVCV proteins (SVCV-N, SVCV-P, SVCV-M, SVCV-L, and SVCV-G) consistently suppressed the expression of *Ci*IFNa and antiviral genes in the EPC cells. Viral proteins are not classical PAMPs that can be sensed by PRRs but rather recognized by host antibodies. Within the cells, it is expected that these viral components would interact with host factors to abrogate the production of antiviral effectors to overcome cellular defense. For instance, the SVCV P protein is a decoy substrate for host phosphokinase TBK1, blocking *ifn* production and promoting SVCV replication in zebrafish (44). Also, the SVCV N protein was shown to inhibit *ifn* production by degrading mitochondrial antiviral signaling protein (MAVS), which is an essential regulator for *ifn* production (46). Taken together, it can be concluded that the expression of type I *ifn*s can be induced by viral nucleotide PAMPs but inhibited by viral proteins.

The interaction of fish type I IFNs with their receptors has not been fully elucidated at the structural level. Fish CRFB1–3 and CRFB5 are equivalent to the mammalian IFNAR2 and IFNAR1, respectively (47). In teleost fish, IFNAR2 has been duplicated into CRFB1 and CRFB2, which have longer intracellular regions than those of IFNAR1/CRFB5 (21). Existing evidence suggests that fish group I and II IFNs activate distinct receptor complexes consisting of CRFB1/CRFB5 or CRFB2/CRFB5 to trigger an antiviral response (34). In zebrafish, CRFB1 and CRFB5 have been shown to be activated by IFNφ1 and IFNφ4 (group I) to exert antiviral effects while CRFB2 and CRFB5 are required for the antiviral activity of IFNφ2 and IFNφ3 (group II). In line with these findings, IFNh, also a member of group I type IFNs, preferentially binds to CRFB1 and CRFB5 (21). In the present study, we showed that *Ci*IFNa (group I) interacts with not only CRFB1 and CRFB5 but also CRFB2, indicating that it engages with all three receptors for signaling. Interestingly, co-IP assay revealed that CRFB1 associates with CRFB2, suggesting that they could form a heterodimer. Whether the formation of CRFB1/CRFB2 dimer can transduce signals requires further investigation. Moreover, *Ci*IFNa appeared to have a higher binding affinity with CRFB5 than that with CRFB1 and CRFB2, in agreement with previous observation in mice where IFNAR1 (equivalent to CRFB5) is the high-affinity receptor for *ifn*-β (KD = ~10 nM) while IFNAR2 (equivalent to CRFB1–3) serves as the low-affinity receptor (KD = 1.7 µM) (20). However, our results are in contrast with the finding in humans where the binding affinity of *ifn*-β with IFNAR1 (KD = 30 nM) is lower than that with IFNAR2 (KD = 0.1 nM) (19).

Type I *ifn* signaling relies on the phosphorylation of STAT1 and STAT2. In some teleost species such as cyprinids, two *stat1* genes (STAT1a and STAT1b) are present (35). Fish STAT1a is evolutionarily closer to mammalian STAT than STAT1b and possesses the conserved domains for *ifn* signaling. In contrast, fish STAT1b lacks the C-terminal transcriptional activation domain, acting as a brake to balance the excessive *ifn* activities that may be detrimental to the host cells (35). Previous studies have shown that overexpression of STAT1a increases the *ifn*-induced ISG expression and inhibits viral replication, while STAT1b has opposite effects (32). We found that *Ci*IFNa induced the phosphorylation of STAT1a but had no effect on the phosphorylation level of STAT1b **(** **Figure 8** **)**, providing further support on the opposite roles of STAT1a and STAT1b in fish.

Because the wild-type and mutant without mutation to the key site can activate the antiviral pathway, the cells entered the antiviral state early, which can resist the infection of SVCV. Therefore, by detecting viral proteins SVCV-G and SVCV-N and two genes in the antiviral pathway, it was found that the antiviral effects of E103A and H165A were significantly reduced. The regulation of *mx* and *viperin* was also significantly weakened. Therefore, we speculate that these two sites (Glu103, His165) are the key sites for *Ci*IFNa to exert its antiviral function. Similarly, we further verified it by plaque formation assay and found the same results. We can conclude that Glu103 and His165 are essential for *Ci*IFNa-mediated antiviral activity.

In summary, the crystal structure of grass carp IFNa was solved, and key residues involved in the interaction with its receptors were identified. The *Ci*IFNa (group I *ifn*) was shown to bind to CRFB1, CRFB2, and CRFB5. Structure superposition predicted that Glu103 and Lys117 of *Ci*IFNa are important to the interaction with CRFB5, while Leu17 and His165 are involved in the binding to CRFB1. It has been shown that they are required for the activation of the receptors to phosphorylate STAT1a, and further mutation of Glu103 and His165 decreased the antiviral activity of *Ci*IFNa. Our findings provide insights into the structural evolution of type I IFNs and interaction with receptors in lower vertebrates.

## Data Availability Statement

The crystal structure of grass carp IFNa has been deposited in the NCBI PDB database (http://www.pdb.org) under accession number 7WKH.

## Author Contributions

ZW: investigation, methodology, data curation, and writing—original draft. JX, JF, KW, KC, ZJ, XZhu, WH, XZha, QL, and BW: investigation and methodology. XC: supervision and review and editing. JW and JZ: conceptualization, funding acquisition, project administration, supervision and editing. All authors contributed to the article and approved the submitted version.

## Funding

This work was funded by the National Natural Science Foundation of China (Grant numbers: 32030112 and U21A20268) and Key Laboratory of Marine Biotechnology of Fujian Province (Grant Number: 2021MB01).

## Conflict of Interest

The authors declare that the research was conducted in the absence of any commercial or financial relationships that could be construed as a potential conflict of interest.

## Publisher’s Note

All claims expressed in this article are solely those of the authors and do not necessarily represent those of their affiliated organizations, or those of the publisher, the editors and the reviewers. Any product that may be evaluated in this article, or claim that may be made by its manufacturer, is not guaranteed or endorsed by the publisher.
