# Supplementary material for: Structural and Functional Analyses of Type I IFNa Shed Light Into Its Interaction With Multiple Receptors in Fish
Source: Front Immunol. 2022 Mar 22;13:862764. doi: 10.3389/fimmu.2022.862764 (PMC8980424; doi:10.3389/fimmu.2022.862764)
Supplement: Supplementary file 1 [file DataSheet_1.docx]

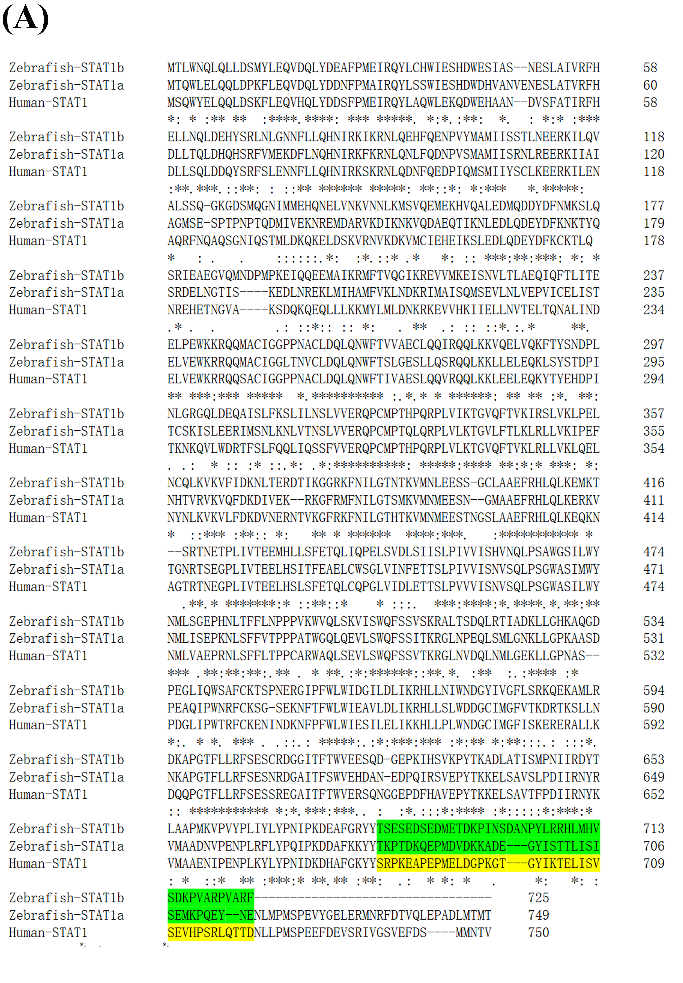


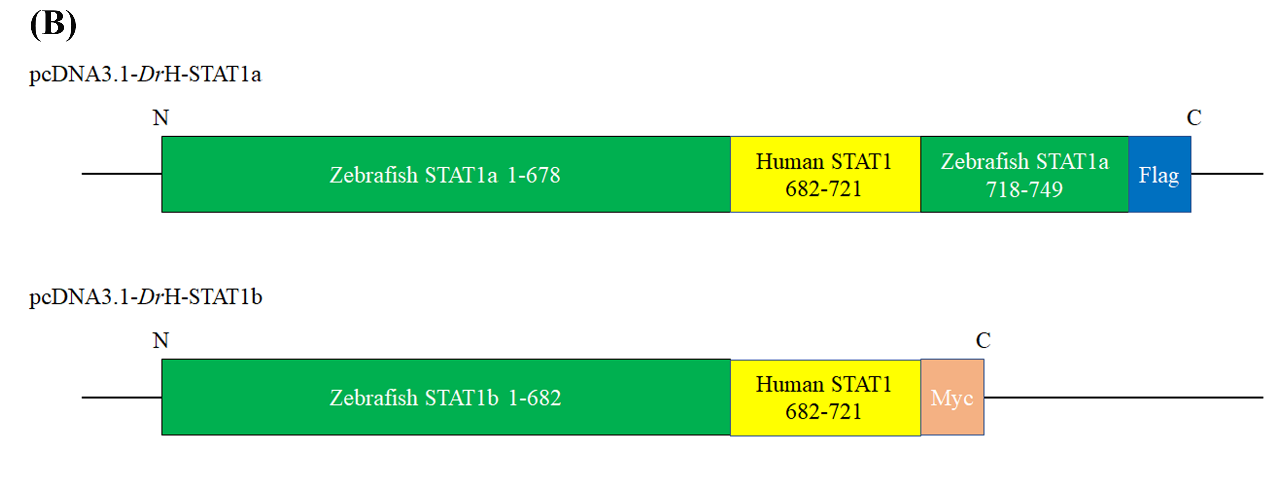


**Supplementary Figure 1** Sequence alignment of zebrafish STAT1a and STAT1b with human STAT1. **(A)**. Diagram description of pcDNA3.1-*Dr*H-STAT1a and pcDNA3.1-*Dr*H-STAT1b plasmid (B). Green indicates zebrafish STAT1a and STAT1b and yellow indicates human STAT1. labeled sequence **(A)**. Diagram description of zebrafish/human pcDNA3.1-*Dr*H-STAT1a and pcDNA3.1-*Dr*H-STAT1b plasmid **(B)**.


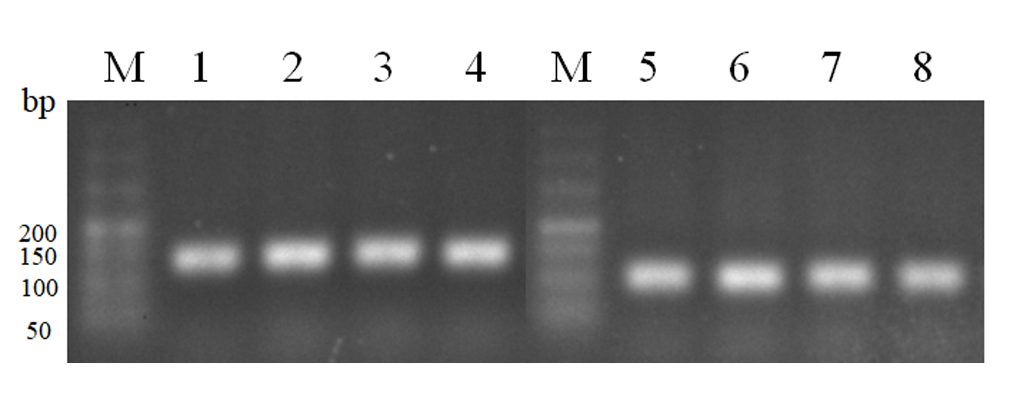


**Supplementary Figure 2.** Expression analysis of *svcv-g*, *svcv-n* in the EPC cells after infection with SVCV. The EPC cells were seeded in 12 well culture plates and infected with SVCV (MOI=0.1). After 24 h, the cells were harvested for PCR analysis of viral gene expression using the primers described in Table 1. M, DNA marker;1-4: SVCV-G, 5-8: SVCV-N. N=4.


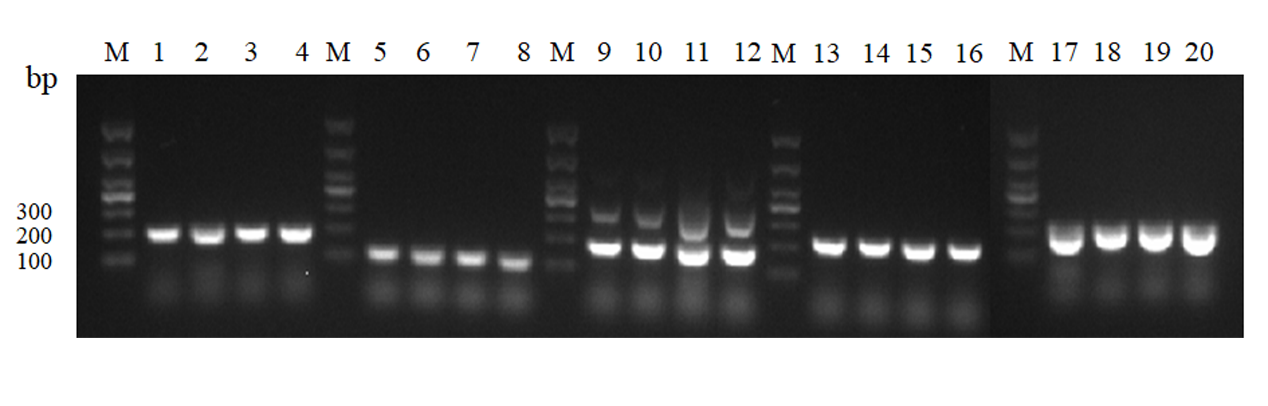


**Supplementary Figure 3.** Expression analysis of viral genes in the EPC cells after transfection with pcDNA3.1-SVCV-N, pcDNA3.1-SVCV-L, pcDNA3.1-SVCV-G, pcDNA3.1-SVCV-M or pcDNA3.1-SVCV-P. The EPC cells were seeded in cell 12 well culture plates and transfected with plasmids using jetPRIME^®^ transfection reagent (Polyplus). After 24 h, the cells were harvested for PCR analysis of viral gene expression using the primers described in Table 1. M, DNA marker;1-4: SVCV-N, 5-8: SVCV-L, 9-12: SVCV-G, 13-16: SVCV-M, 17-20: SVCV-P. N=4.

**
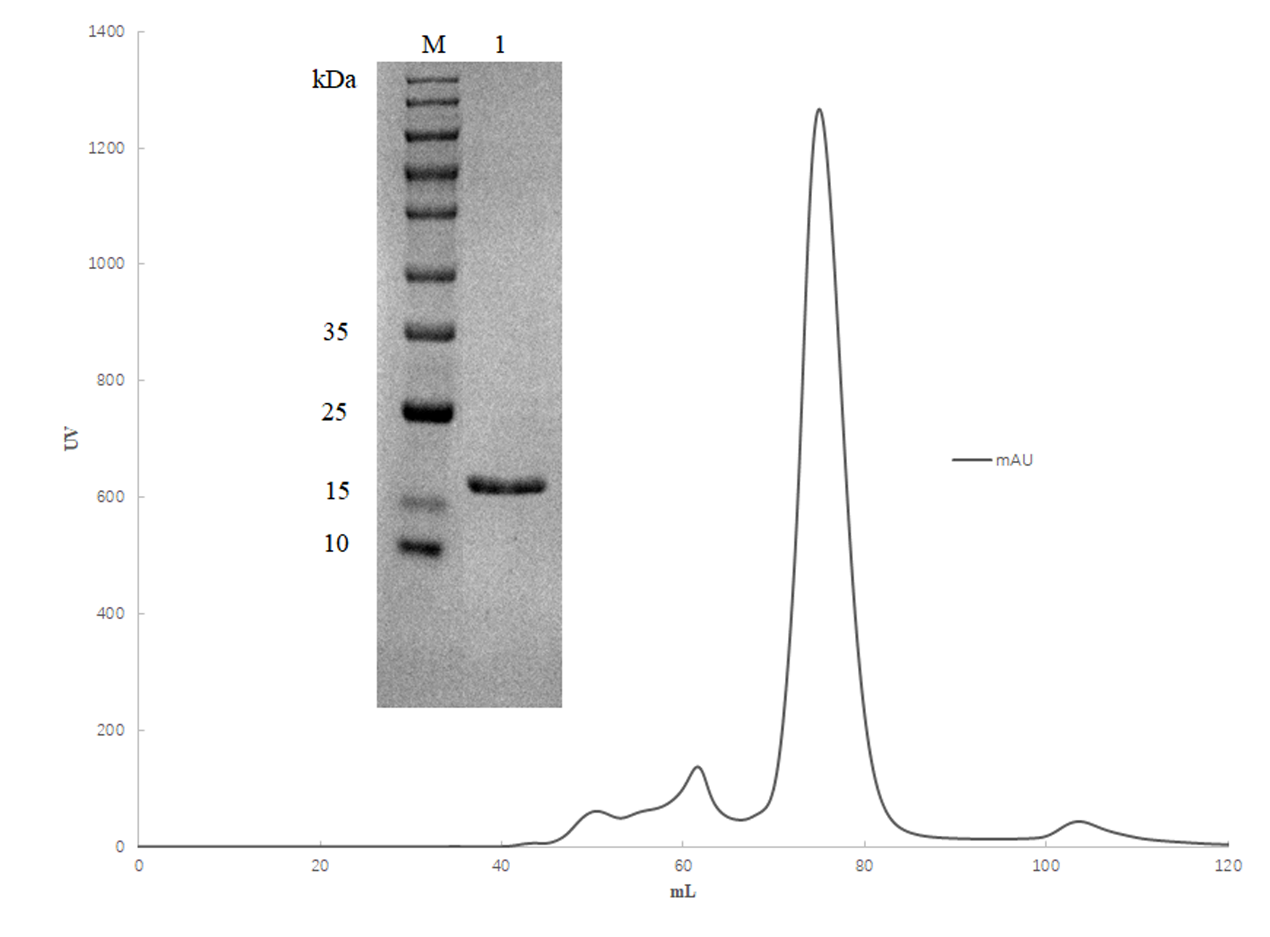
**

**Supplementary Figure 4.** Purification SDS-PAGE analysis of recombinant IFNa protein from bacteria by size exclusion chromatography. M, protein molecular weight marker; 1, purified IFNa.


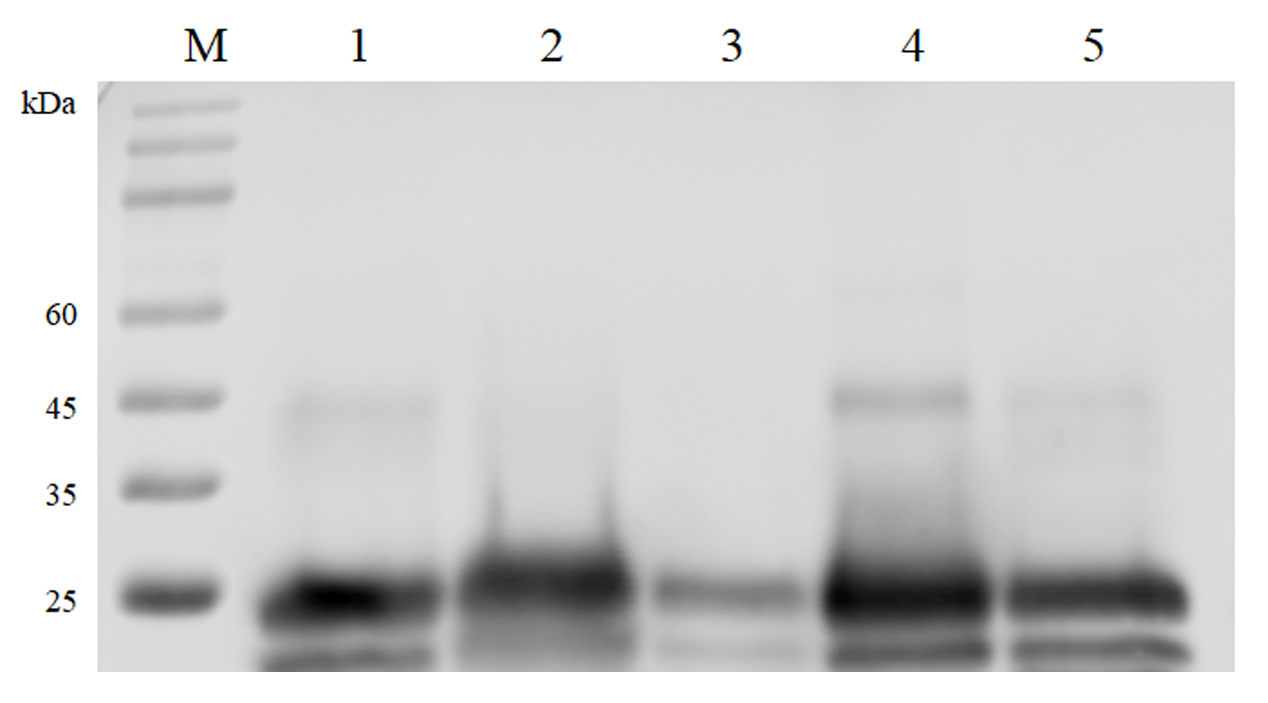


**Supplementary Figure 5.** Validation of recombinant IFNa mutant proteins purified from the HEK293F cells by Western blotting. Anti-6 x His tag antibody was used for Western blotting. M, protein molecular weight marker; 1, wildtype *Ci*IFNa-His; 2, L17A-His; 3, E83A-His; 4, K97A-His; 5, H145A-His.
